# Dietary Counseling: An Option to Malnutrition and Masticatory Deficiency in Patients with Total Protheses? A Scoping Review

**DOI:** 10.3390/nu17010141

**Published:** 2024-12-31

**Authors:** Gloria Cifuentes-Suazo, Josefa Alarcón-Apablaza, Marcela Jarpa-Parra, Camila Venegas, Franco Marinelli, Ramón Fuentes

**Affiliations:** 1 Master Program in Dental Sciences, Dental School, Universidad de La Frontera, Temuco 4781176, Chile; franco.marinelli@ufrontera.cl; 2 Department of Morphology, Faculty of Medicine, Faculty of Dentistry, Universidad Nacional Andrés Bello, Concepción 4260000, Chile; 3 Doctoral Program in Morphological Sciences, Faculty of Medicine, Universidad de La Frontera, Temuco 4781176, Chile; josefa.alarcon@ufrontera.cl; 4 Research Centre in Dental Sciences (CICO-UFRO), Dental School, Universidad de La Frontera, Temuco 4781176, Chile; camilabelen.venegas@ufrontera.cl; 5 Natural Resources and Polymers Research Laboratory, Universidad Adventista de Chile, Chillán 3780000, Chile; marcelajarpa@unach.cl; 6 Department of Integral Adult Dentistry, Dental School, Faculty of Dentistry, Universidad de La Frontera, Temuco 4781176, Chile

**Keywords:** diet, nutritional intervention, oral health, dentures and nutrition, chewing efficiency, nutritional guidance, nutrition counseling, edentulism

## Abstract

Given the rising global population of older adults and their association with edentulism and the use of complete removable prostheses [CRP], it is imperative to pursue solutions for issues such as the relationship between poor diets and masticatory deficiency. Objective: To examine the research on the efficacy of dietary counseling in enhancing mastication and nutrition in older adults with CRP. Methods: A systematic literature review was performed in the PubMed, Trip, and Web of Science databases. Results: 812 results were retrieved from the databases, from which 6 clinical studies that fulfilled the qualifying criteria were selected. The selected studies reported reduced nutrition in patients with CRP due to impaired masticatory function. Research suggests that employing basic dietary guidelines or simplified nutritional recommendations enhances the masticatory function of patients with CRP, thereby ensuring sufficient nutritional intake. Conclusion: Dietary counseling improves nutritional intake and masticatory function in patients with CRP. This would allow simple dietary advice to be given to patients with total prostheses in clinical practice. Further randomized clinical trials are recommended to increase the available evidence.

## 1. Introduction

Edentulism and limitations in oral rehabilitation are pivotal elements that profoundly influence the health and well-being of older adults, with repercussions evident in both the oral cavity and overall health status. Key issues related to these diseases include dissatisfaction with dental prostheses, malnutrition, and reduced masticatory function, collectively resulting in a decline in quality of life [1]. This review will address the effects of malnutrition and loss of masticatory function in this population.

The World Health Organization [WHO] reports that in 2020, the proportion of the global population aged over 60 exceeded that of children under five. By 2030, one in six people globally will be 60 or older, and by 2050, the population over 60 is expected to double, with those over 80 tripling during the same period [2]. As the aging population grows, so does the prevalence of edentulism. According to the WHO Global Oral Health Status Report, 6.82% of the global population is edentulous, with nearly 23% of individuals over 60 affected [3]. The 2021–2030 period has been designated as the Decade of Healthy Aging, focusing on mitigating health inequalities [2].

Tooth loss directly influences food selection and preference, causing denture patients to favor less nutritious soft foods [4]. Edentulous individuals experience a marked reduction in masticatory strength and ability relative to those with complete natural dentition [5]. These dietary changes typically occur after the insertion of new prostheses as professionals advise patients to consume soft foods during the first days after insertion, and this frequently becomes a habit [6,7]. These patients avoid raw fruits and vegetables and fibrous meats, reducing their nutritional intake [8].

Oral health-related quality of life [OHRQoL] in adults is significantly affected by tooth loss, and the degree of deterioration correlates with the number, location, and distribution of missing teeth [9,10]. Edentulism alters eating habits and affects interpersonal communication, self-esteem, and overall quality of life [11]. In addition, increasing evidence associates edentulism with the onset of multiple systemic diseases, such as cardiovascular disease, neurodegenerative disorders, cognitive impairment, and depression [12,13,14,15,16]. Müller et al. and Techapiroontong et al. have shown that quality of life is more affected in users of complete removable prostheses [CRP] [17,18]. Nanri et al. demonstrated that greater fruit and vegetable consumption is strongly associated with a positive OHRQoL in older adults [19].

Masticatory dysfunction, either from periodontal disease, tooth extraction, or the intake of soft foods, greatly impacts higher brain processes, including memory and learning [20]. A new hypothesis postulates that changes in dental functionality and masticatory apparatus may cause brain damage resulting from altered brain circulation and dysfunctional homeostasis [21]. Masticatory function may increase cortical blood flow, activating key brain areas such as the somatosensory area, supplementary motor area, insular lobe, striatum, thalamus, and cerebellum. This increase in cerebral blood flow could positively affect a wide range of cognitive tasks [22,23].

Evidence shows a bidirectional relationship between decreased masticatory function and malnutrition in edentulous patients, attributed to their preference for soft and/or ground food, which hinders sufficient nutrient intake. Maintaining the oral functional health of older adults could be crucial to preventing malnutrition in this population [5,24]. In addition to the nutritional deficiency present in most of the edentulous elderly population with CRP, this condition correlates with impaired brain functions, worsened by the absence of chewing hard foods in favor of ground food.

Despite the extensive evidence linking edentulism and masticatory dysfunction to systemic health and quality of life, there remains a notable gap in the literature regarding effective interventions to mitigate these issues in older adults using complete removable prostheses (CRP). Dietary counseling, as a potential strategy to address malnutrition and improve masticatory performance, has not been systematically evaluated in this specific population. Considering the aging global demographic and the increasing prevalence of edentulism, exploring and synthesizing existing evidence on this topic are essential to inform clinical practices and public health strategies. This review is particularly relevant as it aims to investigate the evidence concerning the potential of dietary counseling to improve mastication and nutrition in older adults with CRP.

## 2. Materials and Methods

### 2.1. Search Strategy

A scoping review was performed that included articles that analyzed whether dietary counseling helps improve mastication and nutrition in older adults with CRP. This review follows the guidelines of the Preferred Reporting Items for Systematic Reviews and Meta-Analyses extension for Scoping Reviews [PRISMAScR] [25].

An electronic literature search was performed in the Pubmed database (Table S1). The method was modified for inclusion in the Trip Database and Web of Science. In addition, the literature was searched manually, and the references in the articles found in the electronic database were reviewed. The terms selected for the search were los siguientes: “Mouth, Edentulous”, “Jaw, Edentulous”, “Dental Prosthesis”, “prosthetic rehabilitation”, “complete denture*”, “Prosthesis, Dental”, “Prostheses, Dental”, “Dental Prostheses”, “Toothless Mouth”, “Edentulous Mouth*”, Edentulous, “Nutrition Therapy”, “Nutrition Assessment”, “Recommended Dietary Allowances”, “Diet Therapy”, “Mini nutritional assessment”, “dietary intervention”, “dietary advice”, “Medical Nutrition Therapy”, OR, “Recommended Dietary Allowances”, “Nutritional Status”, “Diet”, “nutrient intake”, “nutritional intake”, “nutrition”, “malnutrition”, “Diet”, “Mastication”, “Bite Force”, “Bite Forces”, “Masticatory Forces”, “Chewing”, “masticatory function”, ”chewing capacity” (Table S2). The keywords were combined with Boolean OR and AND operators. The search was conducted between March and June 2024.

### 2.2. Eligibility Criteria

Randomized clinical trials were included that examined whether dietary counseling helps improve mastication and/or nutrition in older adults with CRP. The analysis included full-text articles published in the last 10 years, written in English or Spanish. Comparative studies with dental implants, studies of Asian origin with no English translation, and articles where the study group were patients with partial prostheses or implants were excluded.

### 2.3. Article Selection and Data Extraction

Two independent reviewers analyzed the articles from the systematic search by reviewing titles and abstracts. Articles that met the eligibility criteria were analyzed in the full text to confirm their relevance. In cases of disagreement between the two reviewers, a third reviewer was invited to help resolve differing opinions.

The following information was collected from the full-text articles comprising the final selection: authors, years of publication, country, age of study population, and main conclusion of the study. The table used for data extraction was designed by the authors of this review to obtain data relevant to the topic.

## 3. Results

### 3.1. Study Selection

**Figure 1:**
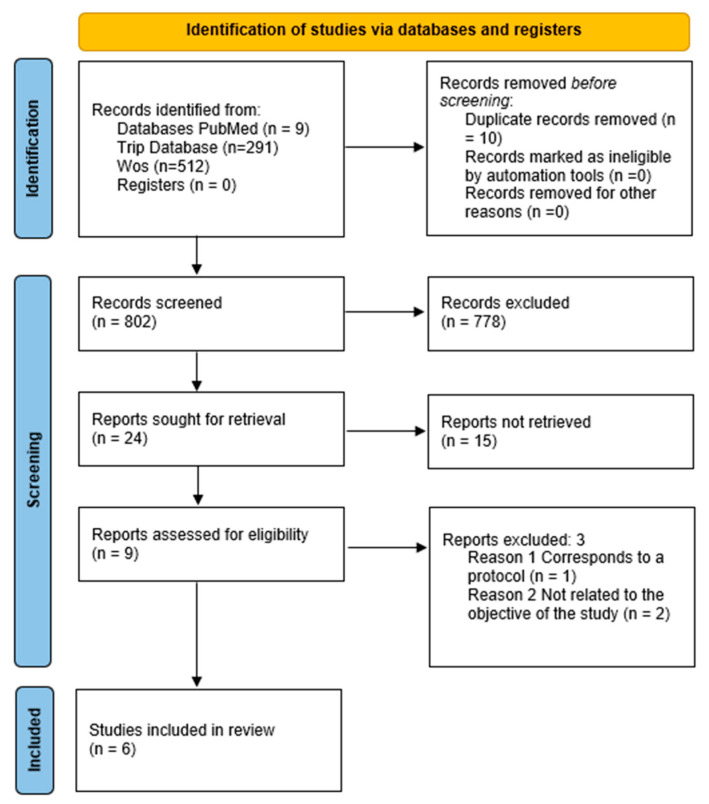
PRISMA 2020 flow chart for new systematic reviews that included only database and registry searches.

Figure 1 illustrates the article search and selection process. Eight hundred and twelve articles were identified in the metasearch engines. Once duplicate files had been eliminated.

Following the preliminary title review, 778 articles were eliminated due to their irrelevance to the study objectives and/or met the exclusion criteria, 473 articles discussed implant versus full denture rehabilitation, 122 addressed bone regeneration, 53 explored dental adhesives, 21 examined different types of prosthetic rehabilitations, 32 discussed dental hygiene and education performed on patients with full dentures, and in 77 articles the comparison group was partial dentures. Subsequently, 15 studies were discarded because they were unrelated to the study topic, or the design did not meet the inclusion criteria. After reading the full-text articles (nine articles), three were excluded for being unrelated to the study objective or for being a protocol. Finally, this study included six articles about randomized clinical trials that examined the impact of dietary counseling on mastication and nutrition in older adults with CRP [26,27,28,29,30,31].

### 3.2. Characteristics of the Selected Studies

The randomized clinical trials that examined whether dietary counseling helps improve mastication and nutrition in older adults with CRP selected in this review are described in Table 1.

**Figure 2:**
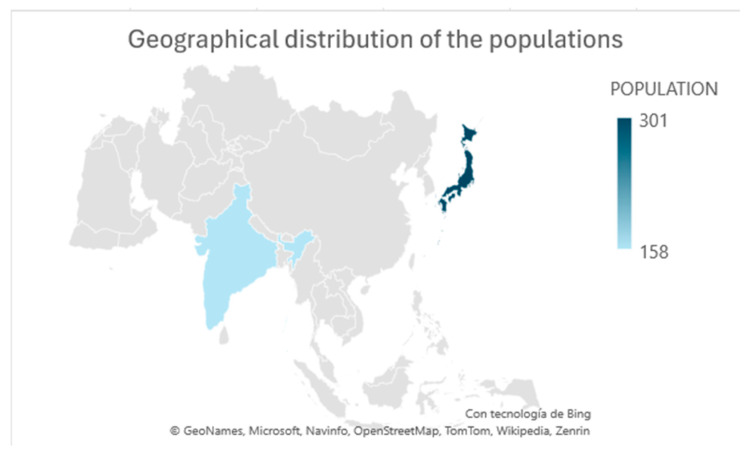
Geographical distribution of the populations included in the selected studies.

All included papers are randomized clinical trials; the articles contained in this review are in English and originate from Japan and India (Figure 2).

Clinical trials collectively highlight the benefits of integrating dietary advice with CRP to improve masticatory function and/or nutritional outcomes. Five of the clinical trials examine oral rehabilitation using CRP with nutritional advice and its relationship to masticatory function and/or nutritional intake or status [26,27,28,29,30]. Denture fabrication alone was reported to not improve nutritional status; however, the addition of dietary advice significantly reduced malnutrition in the intervention group [28,30]. Manuba Kanazawa et al. [28] found that combining CRP fabrication with nutritional advice increased nutrient intake in edentulous older adults, although the effect appeared to be short-lived. However, if dietary advice lasted for at least three months, participants showed a marked increase in protein and fat intake, ref. [26] as well as increased consumption of nutrient-rich foods such as chicken and vegetables [29]. In addition, masticatory function was shown to improve significantly [29]. Improvements in the ability to mix and cut food are described at both 3 and 6 months [27]. Kapila Kumar et al. [31] explored dietary supplements without dietary advice in edentulous women with CPA. While they observed improvements in bone density, electromyography, and masticatory performance, the absence of dietary advice was identified as a key factor behind the lack of significant changes in nutritional intake [31]. These findings highlighted the critical role of dietary advice in improving the efficacy of oral rehabilitation for edentulous patients.

Despite these promising findings, common limitations were observed in the studies, including the advanced expertise of prosthodontists and the inclusion of independent older adults, which restricts applicability in other settings. They also recommend standardizing measurements of masticatory function and nutritional status [26,27,28,30].

### 3.3. Risk of Bias

**Figure 3:**
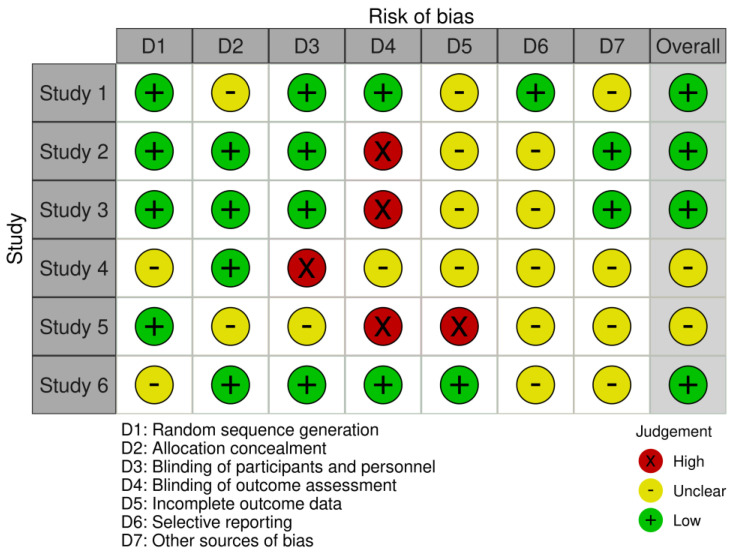
Risk of bias. Study 1 = Hiroyuki Suzuki et al., 2019 [27]. Study 2 = Manuba Kanazawa et al., 2019 [28]. Study 3 = Hiroyuki Suzuki et al., 2017 [26]. Study 4 = Noriko Amagai et al., 2017 [29]. Study 5 = Hiroyuki Suzuki et al., 2018 [30]. Study 6 = Kapila Kumar et al., 2023 [31].

**Figure 4:**
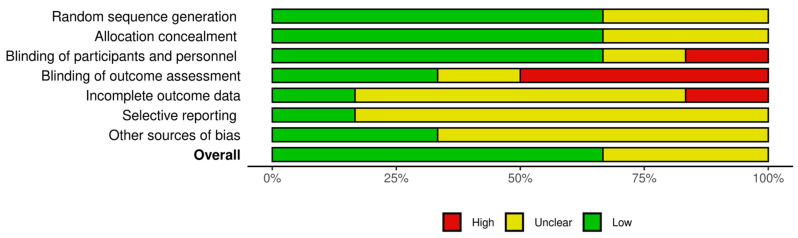
Risk of bias.

The risk of bias was assessed according to The Cochrane Collaboration’s tool for assessing risk of bias in randomized trials [32]; seven domains are assessed for each of the clinical trials included in the review: random sequence generation (selection bias), allocation concealment (selection bias), blinding of participants and researchers (performance bias), blinding of outcome assessment (detection bias), incomplete outcome data (attrition bias), selective reporting (reporting bias), and other bias. The Robvis [33] tool was used to design the graphical representation of the risk of bias, Figure 3 and Figure 4.

## 4. Discussion

The findings in this analysis align with the systematic review by McGowan et al., which indicates that the evidence endorses dietary intervention in conjunction with oral rehabilitation [34]. Their systematic review emphasizes nutritional status and does not address masticatory function.

A 2008 randomized clinical trial [35] determined that prosthetic rehabilitation alone could not sustainably improve patients’ nutritional status since masticatory capacity and efficiency are not the sole determinants. In addition, nutrition is not only a matter of mastication, but also depends on other factors such as habits, tastes, and customs [33]; therefore, counseling may be a way to narrow these gaps.

Extensive evidence indicates that CRP markedly enhance quality of life; however, the mere fabrication of a CRP is insufficient to ameliorate nutritional status. Even simple dietary counseling proves to be effective in achieving better outcomes [30]. The studies reviewed concluded that simple dietary advice coupled with the fabrication of CRP can lead to early improvements in masticatory function [27] and consequently increase the nutrient intake of edentulous older adults [26,27,28,29]. Hiroyuki Suzuki [26], Noriko Amagai [29], and colleagues proved that following three months of a new CRP insertion in addition to dietary counseling, there was a significant increase in the intake of protein, as well as lipids, sodium, potassium, magnesium, phosphorus, iron, zinc, vitamin B2, vitamin B6, niacin, folic acid, and pantothenic acid [26]. Several micronutrients, such as B vitamins and iron, as well as many polyphenols, play a crucial role in cognitive health, and their consumption results in a lower risk of cognitive impairment and dementia [36]. Therefore, a new CRP [26] and mainly dietary counseling [26,27,28,29] strongly impact the intake of nutrients that could prevent cognitive decline and improve older adults’ quality of life. Similarly, the literature shows that mastication is a protective factor for cognitive impairment [22,37]. However, the improvement in nutrient intake in older edentulous patients due to basic dietary counseling may be transient; therefore, the consistent application of these interventions in dental practice is advised to prolong their efficacy [28].

Limitations of the review include the availability of randomized clinical trials. In addition, we encountered unclear risk of bias present in two of the studies [26,29]; however, the predominance of randomized clinical trials with low risk of bias strengthens confidence in the results, while the critical analysis of the trials included in the review with unclear risk of bias allowed us to contextualize the results within the overall evidence framework. The decision not to eliminate any studies strengthens the review by avoiding publication and/or reporting bias.

Despite the little evidence regarding the topic under consideration, the implementation of dietary advice presents few challenges, can be integrated into routine dental practice, poses no risks, and, conversely, offers benefits to patients.

Future trends in this area of research consider an interdisciplinary approach between nutritionists and dentists, which would allow better management and treatment of patients with total prostheses. Along the same lines, it would be possible to work on personalized nutritional counseling using digital tools that facilitate communication and work between health professionals.

To obtain better results, it is necessary to study oral physiology, including masticatory biomechanics, to improve the standardization of the studies and, consequently, the results obtained.

## 5. Conclusions

The evidence reveals a trend that dietary counseling can improve nutritional intake and masticatory function in patients with CRP.

It is suggested that measurement protocols for the assessment of masticatory function be standardized and that more randomized clinical trials be conducted to increase the available evidence.

## Figures and Tables

**Table 1:** Randomized clinical trials that examined whether dietary counseling helps improve mastication and nutrition in older adults with CRP.

Authors	Countries	Study Population	Age	Main Conclusion
Hiroyuki Suzuki et al., 2017 [26].	Japan	62 participants. - 15 men and 16 women in the control group. 16 men and 15 women in the intervention group.	Mean age of control group: 78.6 ± 6.6 years. Mean age of intervention group: 75.3 ± 8.2 years. Mean age: 77 ± 7.6 years.	Simple dietary advice using a uniform leaflet and a new fabrication of CRP increased nutrient intake and masticatory function in healthy, edentulous older adults.
Hiroyuki Suzuki et al., 2019 [27].	Japan	59 participants: - 15 men and 15 women in the intervention group. 15 men and 14 women in the control group.	Mean age of intervention group: 74.8 ± 8 years. Mean age of control group: 78.6 ± 6.8 years. Mean age: 76.7 ± 7.6 years.	Simple dietary advice could lead to relatively early improvements in masticatory function.
Manuba Kanazawa et al., 2019 [28].	Japan	59 participants: - 15 men and 15 women in the intervention group. 15 men and 14 women in the control group.	Mean age of intervention group: 74.8 ± 8 years. Mean age of control group: 78.6 ± 6.8 years. Mean age: 76.7 ± 7.6 years.	The new fabrication of CRP with simple dietary advice could improve nutrient intake in older edentulous patients; however, the effect will likely be short-term.
Noriko Amagai et al., 2017 [29].	Japan	62 participants. - 15 men and 16 women in the control group. 16 men and 15 women in the intervention group.	Mean age of control group: 78.6 ± 6.6 years. Mean age of intervention group: 75.3 ± 8.2 years. Mean age: 77 ± 7.6 years.	Simple dietary advice combined with full prosthesis treatment could improve food intake in edentulous patients.
Hiroyuki Suzuki et al., 2018 [30].	Japan	59 participants. - 15 men and 14 women in the control group. 15 men and 15 women in the intervention group.	Mean age of control group: 78.6 ± 6.8 years. Mean age of intervention group: 74.8 ± 8 years. Mean age: 76.7 ± 7.6 years.	The nutritional status of the healthy, edentulous elderly population could be improved by fabricating new CRP and providing simple dietary advice.
Kapila Kumar et al., 2023 [31].	India	158 participants - 56 women in the intervention group with food supplements. - 50 women in the intervention group with food supplements. 52 women in the control group.	Mean age of the intervention group with food supplements: 50.5 ± 8.85 years. Mean age of intervention group without food supplements: 51.3 ± 7.9 years. Mean age of control group: 49.6 ± 7.95 years.	A statistically significant change in electromyography and masticatory performance was observed in the supplemented group.

## Data Availability

The data presented in this study are available on request from the corresponding author. The data are not publicly available due to no public database is available.

## Supplementary Materials

The following supporting information can be downloaded at: https://www.mdpi.com/article/10.3390/nu17010141/s1, Table S1. Final Pubmed search strategy. Table S2. Search strategy performed in Pubmed based on Patient-Intervention-Results.
