# Supplementary material for: Dietary Counseling: An Option to Malnutrition and Masticatory Deficiency in Patients with Total Protheses? A Scoping Review
Source: Nutrients. 2024 Dec 31;17(1):141. doi: 10.3390/nu17010141 (PMC11723342; doi:10.3390/nu17010141)
Supplement: Supplementary file 1 [file nutrients-17-00141-s001.zip › nutrients-3392551-supplementary.pdf]

Table S1. Final Pubmed search strategy.

Search strategy	**[[[[[[[[[[["Mouth, Edentulous"[Mesh] OR "Jaw, Edentulous"[Mesh]] OR ["Dental Prosthesis"[Mesh]]] OR ["prosthetic rehabilitation"]] OR ["complete denture*"]] OR ["Prosthesis, Dental"]] OR ["Prostheses, Dental"]] OR ["Dental Prostheses"]] OR ["Edentulous Jaw*"]] OR ["Toothless Mouth"]] OR ["Edentulous Mouth*"]] OR [Edentulous]] AND [[[[[[[[[[[[[[["Nutrition Therapy"[Mesh]] OR ["Nutrition Assessment"[Mesh]]] OR ["Recommended Dietary Allowances"[Mesh]]] OR ["Diet Therapy"[Mesh]]] OR ["Mini nutritional assessment"]] OR ["dietary intervention"]] OR ["dietary advice"]] OR ["Therapy, Nutrition"]] OR ["Medical Nutrition Therapy"]] OR ["Therapy, Medical Nutrition"]] OR ["Assessment*, Nutrition"]] OR ["Nutrition Assessment*"]] OR ["Assessment*, Nutritional"]] OR ["Diet Therapies"]] OR ["Therapy, Diet"]] OR [Recommended Dietary Allowances]]] AND [[[[[[["Nutritional Status"[Mesh]] OR ["Diet"[Mesh]]] OR ["nutrient intake"]] OR ["nutritional intake"]] OR [nutrition]] OR [malnutrition]] OR ["Diet"]] OR [[[[[["Mastication"[Mesh]] OR ["Bite Force"[Mesh]]] OR ["Bite Forces"]] OR ["Masticatory Forces"]] OR ["Chewing"]] OR ["masticatory function"]] OR ["chewing capacity"]]]**

Table S2. Search strategy performed in Pubmed based on Patient-Intervention-Results.

		Free term	Mesh	Search method
Patients	Edentulism	• Edentulous<br>• "Edentulous Mouth*"<br>• Toothless Mouth<br>• Edentulous Jaw*<br>• Dental Prostheses<br>• Prostheses, Dental<br>• Prostheses, Dental<br>• "complete denture*"<br>• prosthetic rehabilitation	"Mouth, Edentulous"[Mesh]<br>"Jaw, Edentulous"[Mesh]<br>"Dental Prosthesis"[Mesh]	**[[[[[[[[[["Mouth, Edentulous"[Mesh] OR "Jaw, Edentulous"[Mesh]] OR ["Dental Prosthesis"[Mesh]]] OR ["prosthetic rehabilitation"]] OR ["complete denture*"]] OR ["Prosthesis, Dental"]] OR ["Prostheses, Dental"]] OR ["Dental Prostheses"]] OR ["Edentulous Jaw*"]] OR ["Toothless Mouth"]] OR ["Edentulous Mouth*"]] OR [Edentulous]**
Intervention	Dietary counseling	• "Mini nutritional assessment"<br>• "dietary intervention"<br>• "dietary advice"<br>• Therapy, Nutrition<br>• Medical Nutrition Therapy<br>• Therapy, Medical Nutrition<br>• Assessment*, Nutrition<br>• Nutrition Assessment*<br>• Assessment*, Nutrition	• "Nutrition Therapy"[Mesh]<br>• "Nutrition Assessment"[Mesh]<br>• "Recommended Dietary Allowances"[Mesh]<br>• **"Diet Therapy"[Mesh]**	**[[[[[[[[[[[[[[["Nutrition Therapy"[Mesh]] OR ["Nutrition Assessment"[Mesh]]] OR ["Recommended Dietary Allowances"[Mesh]]] OR ["Diet Therapy"[Mesh]]] OR ["Mini nutritional assessment"]] OR ["dietary intervention"]] OR ["dietary advice"]] OR ["Therapy, Nutrition"]] OR ["Medical Nutrition Therapy"]] OR ["Therapy, Medical Nutrition"]] OR ["Assessment*, Nutrition"]] OR ["Nutrition Assessment*"]] OR ["Assessment*, Nutritional"]] OR ["Diet**

		• Diet Therapies<br>• Therapy, Diet		**Therapies"]] OR ["Therapy, Diet"]] OR [Recommended Dietary Allowances]**
Result:	Malnutrition and/or	• nutrient intake<br>• nutritional intake<br>• nutritional condition<br>• nutrition<br>• malnutrition<br>• Diet	• Nutritional Status"[Mesh]<br>• "Diet"[Mesh]	**[[[[["Nutritional Status"[Mesh]] OR ["Diet"[Mesh]]] OR ["nutrient intake"]] OR ["nutritional intake"]] OR [nutrition]] OR [malnutrition]] OR ["Diet"]**
	Masticatory deficiency	• Bite Forces<br>• Masticatory Forces<br>• Chewing<br>• masticatory function.<br>• chewing capacity	• "Mastication"[Mesh]<br>• "Bite Force"[Mesh]	**[[[[["Mastication"[Mesh]] OR ["Bite Force"[Mesh]]] OR ["Bite Forces"]] OR ["Masticatory Forces"]] OR ["Chewing"]] OR ["masticatory function"]] OR ["chewing capacity"]**
